# Root Growth as an Early Indicator of PFAS Phytotoxicity in Plants

**DOI:** 10.3390/toxics14060455

**Published:** 2026-05-22

**Authors:** Lara Nigro, Lorenzo Federico, Valeria Tatangelo, Sara Villa

**Affiliations:** Department of Earth and Environmental Sciences DISAT, University of Milano-Bicocca, Piazza della Scienza 1, 20126 Milano, Italy; lara.nigro@unimib.it (L.N.); valeria.tatangelo@unimib.it (V.T.); sara.villa@unimib.it (S.V.)

**Keywords:** PFCAs, phytotoxicity, germination, root elongation, terrestrial plants, *Sinapis alba*, *Cucumis sativus*, early-growth endpoints, soil ecotoxicology

## Abstract

Perfluoroalkyl carboxylic acids (PFCAs) are persistent contaminants increasingly subjected to regulatory restrictions. To date, their effects on terrestrial plants remain poorly investigated. To address these knowledge gaps, a comparative assessment was conducted to identify the most sensitive plant species and the most responsive early-growth endpoints. Five PFCAs were selected according to their carbon-chain length (from 3 to 8 C-atoms). Seven plant species were exposed to a wide range of concentrations (from 0.01 up to 100 µg kg^−1^). Germination and root elongation were evaluated as developmental endpoints to assess both acute and sublethal effects. Across species, germination exhibited weak responses, whereas root elongation appeared to be the most sensitive screening parameter, displaying divergent species-specific patterns. Notably, *Sinapis alba* and *Cucumis sativus* emerged as the most responsive species, although they exhibited opposite responses. While mustard exhibited low-dose root stimulation, cucumber showed root inhibition. Interestingly, species within the same family (Brassicaceae and Cucurbitaceae) showed contrasting sensitivity, suggesting that PFCA phytotoxicity is species-specific rather than driven by taxonomic relatedness. This divergent pattern may be linked to distinct morpho-physiological traits, supporting their use as suitable model organisms for phytotoxicity screening of PFCAs.

## 1. Introduction

Per- and polyfluoroalkyl substances (PFASs) are a large class of synthetic fluorinated compounds widely used in industrial and consumer products, such as non-stick cookware, food packaging, water- and stain-resistant textiles, surface coatings and firefighting foams, due to their hydrophobicity, oleophobicity and chemical stability [1,2]. Their environmental persistence and high mobility have resulted in widespread contamination of environmental matrices, including soil [3,4,5], raising increasing concern for ecosystem and human health [6]. Previous studies have demonstrated their uptake and accumulation in crop plants, often in species- and compound-dependent patterns [7,8]. Several PFASs are already listed as persistent organic pollutants under the Stockholm Convention. Although uncertainties remain, numerous countries worldwide have introduced bans or restrictions on many of these substances since 2009 [2]. However, compiling an exhaustive list is not feasible, as the regulatory landscape is rapidly and continuously evolving.

To date, information on PFASs’ effects on terrestrial plants remains limited, and the most predictive endpoints for risk assessment are still debated. Reported responses and accumulation patterns differ substantially depending on plant species, PFAS physicochemical properties, exposure concentration, and the specific endpoint considered [5,7,9,10]. Moreover, most experimental evidence remains concentrated on a limited number of compounds, particularly perfluorooctanoic acid (PFOA) and perfluorooctane sulfonate (PFOS) [5,7,11], resulting in a fragmented understanding of PFASs’ effects across homologues and chain lengths. For instance, in wheat (*Triticum aestivum*), PFOA exposure affected germination and seedling growth, with low-dose (<0.2 mg kg^−1^) stimulation of root length and high-dose (>800 mg kg^−1^) inhibition of germination, root and shoot growth [12]. In cucumber (*Cucumis sativus*), soil exposure to PFOA at 0.2 and 5 mg kg^−1^ reduced biomass, chlorophyll content and photosynthetic performance, and altered leaf metabolite profiles [13]. In *Arabidopsis thaliana*, PFOA exposure has been reported to affect root growth, chlorophyll content, antioxidant responses and gene expression [14]. More recently, exposure to 100 µmol L^−1^ PFOA or PFOS was shown to affect primary root growth and photosynthetic performance, with molecular evidence suggesting the involvement of auxin- and abscisic acid-related signalling pathways [15].

Overall, these studies indicate that PFASs’ effects on plants depend on the compound tested, plant species, exposure conditions and selected endpoint. However, most available data derive from studies on single species and/or individual PFASs, limiting direct comparisons of plant sensitivity across species and compounds. Therefore, comparing phylogenetically distinct plant species under controlled experimental conditions is essential to identify sensitive model species and reliable early-warning endpoints for PFAS phytotoxicity assessment.

Within this framework, the present study adopts a multi-species experimental design aligned with established plant bioassay standards, primarily based on OECD Guideline 208 [16], for recommendations supporting multi-species testing strategies, and complemented by UNI EN ISO 18763 [17]; for specific operational procedures and calculation approaches, reference is made to the UNICHIM Method 1651 [18].

In order to reduce chemical heterogeneity and minimize confounding variability due to functional groups, the present study focused on a single congeneric class of compounds, perfluoroalkyl carboxylic acids (PFCAs), which differ primarily in carbon-chain length and include compounds of increasing regulatory concern, such as PFOA and long-chain PFCAs listed under the Stockholm Convention. In addition, to contribute to the development of a rapid and effective diagnostic framework for phytotoxicity assessment, multiple early-response endpoints were evaluated across different plant species and exposure concentrations.

With this aim, the phytotoxicity assessment was conducted using five PFCAs selected to represent a gradient of increasing perfluorinated carbon-chain length, different early developmental endpoints, and phylogenetically distinct model plants.

By comparing responses across species and PFCAs, this study aimed to:

(i) Identify interspecific differences in sensitivity during early developmental stages;
(ii) Determine which early-growth endpoints are most sensitive and diagnostically informative for the exposure assessment of PFCAs;
(iii) Evaluate whether the molecular structure of PFCAs modulates phytotoxicity patterns.

## 2. Materials and Methods

### 2.1. Soil Preparation and Stock Solutions

Experimental soils consisted of LUFA 2.2 Speyer standard soil purchased from LUFA Speyer (Speyer, Germany). The properties of this soil include a pH = 5.5 ± 0.2 in 0.01 M CaCl_2_, WHC = 44.5 ± 3.0 g/100 g, C = 1.77 ± 0.2%, N = 0.17 ± 0.02%, texture = 7.3 ± 1.2% clay; 13.8 ± 2.7% silt; and 78.9 ± 3.5% sand. The following PFCAs were tested: perfluoropropanoic acid (PFPrA; C_3_HF_5_O_2_; CAS 422-64-0), perfluorobutanoic acid (PFBA; C_4_HF_7_O_2_; CAS 375-22-4), perfluoropentanoic acid (PFPeA; C_5_HF_9_O_2_; CAS 2706-90-3), perfluoroheptanoic acid (PFHpA; C_7_HF_13_O_2_; CAS 375-85-9), and perfluorooctanoic acid (PFOA; C_8_HF_15_O_2_; CAS 335-67-1). All PFCA analytical standards (95–98% purity) were purchased from Sigma-Aldrich (St. Louis, MO, USA). Individual stock solutions of each compound were prepared in ultrapure water and stored in high-density polyethylene (HDPE) containers to prevent adsorption onto glass [19]. Stock nominal concentrations (20 µg mL^−1^) were selected based on the aqueous solubility of each compound. Prior to use, stock solutions were mixed on a rotary shaker to ensure homogeneity and subsequently diluted in Milli-Q water to prepare working solutions required to achieve the target soil concentrations (0.01, 0.1, 1, 10, and 100 µg kg^−1^ dry weight (d.w.)). This concentration range was selected to cover low-dose soil exposure scenarios and allow the comparison of early plant responses across PFCA homologues. Available data on PFCA concentrations in soils are still limited and highly variable, particularly for compounds other than PFOA. Therefore, PFOA was used as a reference compound to contextualize the selected exposure range, as it is one of the most studied PFCAs and has been reported in soils at background concentrations ranging from 0.01 to 123.6 µg kg^−1^, with levels of up to approximately 50,000 µg kg^−1^ in highly contaminated soils [20].

### 2.2. Species Selection

All selected species are included in the OECD Test Guideline 208 list of species [16]. Specifically, six plant species were selected to represent different taxonomic families: sorghum (*Sorghum bicolor*, Poaceae), watercress (*Lepidium sativum*, Brassicaceae), mustard (*Sinapis alba*, Brassicaceae), cucumber (*Cucumis sativus*, Cucurbitaceae), tomato (*Solanum lycopersicum*, Solanaceae), and lettuce (*Lactuca sativa*, Asteraceae). An additional species, zucchini (*Cucurbita pepo*, Cucurbitaceae), was included to compare responses within Cucurbitaceae and to assess whether the higher sensitivity observed in cucumber reflected a broader family-level susceptibility or a species-specific trait. All seeds were purchased from Franchi Sementi S.p.A. (Grassobbio, BG, Italy) as untreated and non-coated, with a declared purity of 97–98% and a germination rate of at least 75%.

### 2.3. Phytotoxicity Bioassay

Early developmental tests were conducted following the UNI EN ISO 18763 guideline [17] with a minor methodological adaptation from the UNICHIM [18] phytotoxicity protocol regarding the oversaturation of soil, as this is not reported in the ISO guidelines. For each treatment, 10 g d.w. of soil was weighed directly into sterile 90 mm Petri dishes. Each treatment and control were prepared in triplicate (*n* = 3). For each control and PFCA treatment, soils were first adjusted to 100% WHC using distilled water. Subsequently, 5 mL of ultrapure water or contaminated solution was added to each Petri dish in controls and treatments, respectively. All soils were gently homogenized to ensure uniform soil moisture and optimal seed–soil contact. Spiking was performed immediately before seed placement. A circular filter paper disc was placed on the soil surface; above it, ten seeds were placed using tweezers. Petri dishes were sealed with parafilm to minimize evaporation and incubated in a non-ventilated thermostatic chamber ISCO (Permax S.r.l., Milan, Italy) at 25 ± 2 °C in complete darkness. Exposure duration was 72 h for all species except for tomato, which was extended to 7 days in accordance with ISO guidelines [17]. At the end of the exposure period, seedlings from each Petri dish were gently placed adjacent to a metric ruler and photographed using a camera (Trust Teza 4K Ultra HD webcam, 3024 × 4032 pixels, Trust International B.V., Dordrecht, The Netherlands) at a fixed focal distance of 35 cm. Digital images were then analyzed using ImageJ v.1.54 to obtain calibrated morphometric measurements.

### 2.4. Data Collection and Analysis

#### 2.4.1. Germination and Root Length

Morphometric imaging analysis was performed to quantify the radicle protrusion. Seed were considered germinated when the protrusion was ≥1 mm. Root length was then measured for each germinated seed [17]. In addition, the Germination Index (GI%) was calculated according to the UNICHIM approach [18] as an integrated phytotoxicity parameter combining seed germination and root elongation. The GI% results are reported in the Supplementary Information (SI). For each Petri dish, the mean root length (M_LR_) and the maximum root length (M_LLR_) were calculated. The mean (M_LLR_) was calculated as the average of the three M_LLR_ values.

Germination inhibition (G%) was calculated as follows:

G% = ((G__Control_ − G__Treatment_)/G__Control_) × 100 (1)

where G is the mean number of germinated seeds.

Root elongation inhibition (L%) was calculated as follows:

L% = ((M_LR_Control_ − M_LR_Treatment_)/M_LR_Control_) × 100 (2)

#### 2.4.2. Statistical Analysis

Prior to statistical analysis, potential outliers were identified using Grubb’s test in OriginLab and excluded when statistically significant (OriginPro, v. 2024). Statistical analyses were performed separately for each plant species, endpoint and PFCA, with the aim of comparing the concentration of each treatment with its corresponding control within the same species. This approach was adopted because the tested species differed markedly in germination dynamics and early growth patterns, which could confound direct cross-species comparisons in a factorial model. Data distribution and homogeneity of variance were assessed using the Shapiro–Wilk test and Levene’s test, respectively. When the assumptions of normality and homoscedasticity were met, treatment effects were analyzed using one-way analysis of variance (ANOVA), followed by Tukey’s HSD post hoc test. When assumptions were invalidated, the non-parametric Kruskal–Wallis test was applied, followed by Dunn’s multiple-comparison test with Bonferroni correction. Statistical significance was set at *p* < 0.05.

Generalized Additive Models (GAMs) were applied to explore non-linear relationships between the PFCA concentration and M_LR_ in the two most responsive species. Models were fitted separately for each PFAS using a Gaussian error distribution and identity link function. The predictor variable was the log-transformed concentration (log(Concentration + 1)), and smoothing was performed using penalized regression splines (basis dimension k = 3–4). Model performance was evaluated using adjusted R^2^, deviance explained, *p*-values associated with the smooth term, and Akaike’s Information Criterion (AIC). Only PFCAs showing a statistically significant smooth term (*p* < 0.05) were retained for graphical visualization.

The predicted M_LLR_ values were estimated by modelling the relationship between M_LR_ and M_LLR_ in the control group for each species. To assess the consistency of this relationship, the ratio between observed and predicted M_LLR_ values was then calculated, providing a quantitative measure with which to verify the specific relationship between M_LR_ and M_LLR_.

## 3. Results

### 3.1. Overall Results

The phytotoxicity bioassays were considered valid, as seed germination in the negative controls exceeded 70%, in accordance with the ISO 18763 criteria [17]. L% displayed the highest number of significant responses across species compared to G%, highlighting a more pronounced effect on root elongation than on germination. Specifically, sporadic effects on seed vitality were observed in *C. pepo*, *C. sativus*, *S. bicolor* and *S. lycopersicum*. The scattered distribution of these observed effects complicates the establishment of a clear cause–effect relationship between acute biological responses (seed germination) and chemical exposure.

Regarding the sublethal effects on root elongation, *S. alba* and *C. sativus* showed significant responses to nearly all tested PFCAs, while *L. sativa* only reported a reduction in the concentration of PFPeA.

Given the large dataset, only statistically significant effects are summarized in Table 1. Detailed statistical analyses of the measured endpoints at each PFCA concentration are reported in the SI, together with the raw data for germination, mean root length and maximum root length (G, M_LR_ and M_LLR_; Table S1). The Germination Index (GI%) was also calculated and is reported in Table S2 as a complementary integrated phytotoxicity index.

### 3.2. Species with Lower Sensitivity to PFCAs

#### 3.2.1. *Sorghum bicolor*

PFCA exposure induced a very limited toxicological response in sorghum, as a significant effect was only observed for G% following PFOA exposure (F_5,12_ = 3.12; *p* = 0.049). Specifically, germination inhibition increased at 1 µg kg^−1^ d.w. (+39.29%; *p* = 0.037, pairwise comparison with the control; Table 1). Overall, these results indicate that *S. bicolor* showed low sensitivity to PFCA exposure, with effects restricted to a single PFOA concentration and limited to the germination phase, which is in agreement with previous findings on *S. bicolor* [7].

#### 3.2.2. *Lepidium sativum*

In watercress, PFCA exposure did not induce statistically significant effects on any of the evaluated endpoints. Accordingly, *L. sativum* was identified as the least responsive species among those tested.

#### 3.2.3. *Cucurbita pepo*

In zucchini, G% showed a significant increase following exposure to PFPeA (F_5,12_ = 4.09; *p* = 0.020). Specifically, increased germination inhibition was observed at 0.01 µg kg^−1^ d.w. (+45.5%; *p* = 0.020) and 10 µg kg^−1^ d.w. (+40.9%; *p* = 0.040). To the best of our knowledge, these data provide the first evidence of PFCA-induced effects on this species.

#### 3.2.4. *Lactuca sativa*

In lettuce, the L% index showed a significant effect following exposure to PFPeA (F_5,12_ = 6.94; *p* = 0.0029), with a significant decrease detected at 100 µg kg^−1^ d.w. (−18.23%; *p* = 0.032). Overall, lettuce showed a PFCA-specific sensitivity in terms of root elongation, in line with previous studies [7,21,22].

#### 3.2.5. *Solanum lycopersicum*

In tomato, G% showed a significant increase following PFOA exposure (F_5,12_ = 3.41; *p* = 0.038), with increased germination inhibition observed at 100 µg kg^−1^ d.w. (+41.7%; *p* = 0.020, pairwise comparison with the control).

### 3.3. Species with Higher Sensitivity to PFCAs

#### 3.3.1. *Sinapis alba*

**Figure 1:**
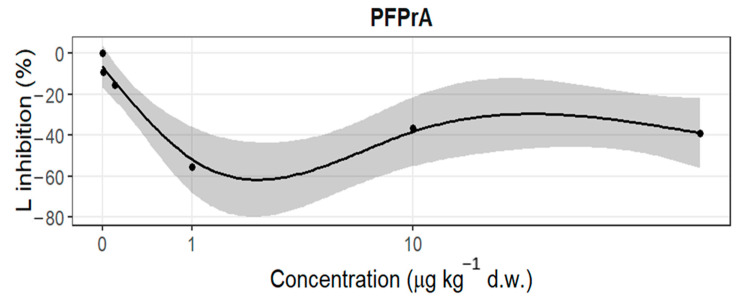
GAM function of PFPrA concentration and root elongation inhibition (L%) in *Sinapis alba*. The solid line represents the fitted smooth function, and the shaded area indicates the 95% confidence interval. Points represent mean observed values at each concentration level (log-transformed as log(Concentration + 1)); k = 4 for the smooth terms.

In mustard, PFCA exposure significantly affected L%, whereas G% remained unchanged across treatments (Figure S1). Within the wide range of concentrations tested (from 0.01 to 100 µg kg^−1^ d.w.), the observed trend revealed a decrease in L%, suggesting a PFCA-dependent stimulatory effect on root growth. However, no role can be assigned to C-length. In fact, a significant decrease in L% was observed in PFPrA exposure (F_5,12_ = 5.24; *p* = 0.008), with inhibition at 1 µg kg^−1^ d.w. compared to the control (−55.4%; *p* = 0.010). Similarly, PFBA affected L% (F_5,12_ = 3.92; *p* = 0.020), with a significant decrease at 0.01 µg kg^−1^ d.w. (−27.2%; *p* = 0.020). PFHpA also affected L% (F_5,12_ = 4.66; *p* = 0.014), with significant decreases at 0.01 µg kg^−1^ d.w. (−40.7%; *p* = 0.009) and 10 µg kg^−1^ d.w. (−36.2%; *p* = 0.020). No statistically significant effects were observed for PFPeA and PFOA. GAM analysis confirmed significant non-linearity only for PFPrA (R^2^ = 0.599; deviance explained = 66.6%; *p* = 0.0017; Figure 1), with an expected inhibition on more than 50% of root elongation at around 1 µg kg^−1^ d.w., whereas no significant non-linearity emerged for the other PFCAs (Table 2), confirming the PFCA-dependent stimulatory effects on *S. alba*.

#### 3.3.2. *Cucumis sativus*

In cucumber, PFCA exposure mainly affected L%, whereas effects on germination were more limited (Figure S2). Among the tested compounds, PFPrA, PFPeA, and PFHpA induced the most evident responses, showing a concentration-dependent reduction in root elongation.

PFPrA significantly affected G% (χ^2^ = 17.26, df = 5, *p* = 0.004), with germination inhibition increasing by 12.5% at 100 µg kg^−1^ d.w. compared with the control (*p* = 0.005). PFPrA also significantly affected L% (F_5,12_ = 3.96; *p* = 0.010), with a significant increase detected at 0.1 µg kg^−1^ d.w. (+26.7%; *p* = 0.021). PFPeA induced significant effects on L% (F_5,12_ = 5.44; *p* = 0.002), with a significant increase at 100 µg kg^−1^ d.w. (+25.1%; *p* = 0.038; Table 1). Exposure to PFHpA produced the strongest response on L% (F_5,12_ = 15.12; *p* < 0.0001), with significant increases observed from 1 to 100 µg kg^−1^ d.w. (40.2–51.3%; 0.001 < *p* < 0.05) (Table 1).

**Figure 2:**
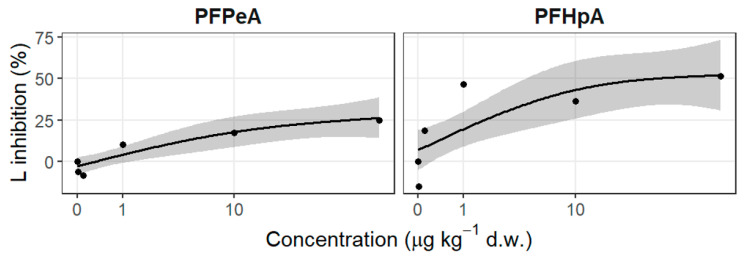
GAMs of concentration and root elongation (M_LR_) due to PFCAs in *Cucumis sativus*. Only PFCAs showing statistically significant smooth terms (*p* < 0.05) are displayed. Solid lines represent fitted smooth functions, and shaded areas indicate 95% confidence intervals. Points represent mean observed values at each concentration level (log-transformed as log(Concentration + 1)); k = 3 for the smooth terms.

GAM analysis further supported this pattern (Figure 2), revealing significant non-linear trends, with the strength of the response in PFPeA (R^2^ = 0.43; deviance explained = 46%; *p* = 0.00058), and PFHpA (R^2^ = 0.47; deviance explained = 52%; *p* = 0.0048) (Table 3). Furthermore, more than 50% of effects on root growth were expected in the range of 10–100 µg kg^−1^ d.w. in PFHpA.

In consideration of the preceding observations, *C. sativus* was identified as the most sensitive species, with detrimental effects on both germination and root growth reported.

### 3.4. Correlation Between M_LR_ and M_LLR_

A positive relationship between M_LR_ and M_LLR_ was observed in all the tested species, suggesting that the two endpoints provide complementary and closely related information, in agreement with the ISO guideline [13]. To evaluate whether M_LLR_ could be used as a reliable proxy endpoint in phytotoxicity screening, a species-specific linear regression was fitted using control data, with M_LLR_ as the dependent variable and M_LR_ as the explanatory variable. These regressions were then used to estimate expected M_LLR_est_ values under treatment conditions. The agreement between observed M_LLR_obs_ and estimated M_LLR_est_ values was assessed by calculating the ratio between observed and predicted M_LLR_ in PFCA-treated samples. This ratio was used to evaluate how much treated samples deviated from the relationship observed under control conditions and, therefore, whether M_LLR_ remained consistent with M_LR_ under PFCA exposure.

As reported in Table 4, observed M_LLR_ values ranged between 80% and 120% of the estimated values across all taxa, although variability differed among species. *S. bicolor* (89% ± 24) and *S. lycopersicum* (90% ± 23) showed a slight tendency toward model overestimation, whereas *L. sativa* showed the highest mean ratio (111% ± 33), indicating greater dispersion and a tendency toward model underestimation. Intermediate values were observed for *C. pepo* (94% ± 25), while *L. sativum* (98% ± 11), *S. alba* (104% ± 11), and *C. sativus* (101% ± 14) showed the closest agreement and lowest variability.

Overall, a good agreement between predicted and observed M_LLR_ values was found across all taxa, supporting the use of M_LLR_ as a reliable and time-efficient endpoint for assessing the phytotoxic effects of PFCAs.

## 4. Discussion

The present study provides the first comparative assessment of PFCAs’ phytotoxicity across multiple plant species and early developmental endpoints, highlighting clear interspecific and compound-specific differences in sensitivity.

### 4.1. Species-Specific Sensitivity to PFCAs

The findings highlighted a high specific sensitivity among the tested plants. Both monocotyledonous and dicotyledonous species exhibited variable responses: *L. sativum* (Brassicaceae) was the only taxon showing no statistically significant variation in the measured endpoints compared with the controls, while *S. bicolor* (Poaceae), *S. lycopersicum* (Solanaceae), *C. pepo* (Cucurbitaceae) and *L. sativa* (Asteraceae) exhibited low responsiveness to PFCAs. In contrast, *S. alba* (Brassicaceae) and *C. sativus* (Cucurbitaceae) showed the highest responsiveness.

Notably, contrasting responses were observed within the same botanical families. Within Cucurbitaceae, *C. sativus* showed marked sensitivity, whereas *C. pepo* remained largely unaffected. Similarly, within Brassicaceae, *S. alba* was highly responsive, while *L. sativum* showed no significant effects. Furthermore, the two most responsive species displayed opposite response patterns, with *S. alba* exhibiting stimulation of root growth and *C. sativus* showing concentration-dependent inhibition. Overall, these findings indicate that PFCA phytotoxicity is highly species-specific and cannot be directly attributed to the botanical family but is more likely driven by differences in physiological traits.

A potential explanation for these responses can be attributed to root system architecture (RSA) as a relevant trait in the effective contact surface between roots and soil matrix [23]. Qualitative visual observations revealed interspecific differences in early root morphology structure (Figure S3). These observations were interpreted in light of previous descriptions of seed germination, early seedling development, root architecture, root hair morphology and broader plant developmental architecture reported in the literature [24,25,26,27,28,29,30,31]. These observations allowed a preliminary classification of the tested species into two root morphological patterns:

(i) Species exhibiting a dense root hair zone with clearly visible root hairs along the primary root axis (*C. sativus* and *S. alba*);
(ii) Species characterized by a single primary root with sparse or poorly visible root hairs (*S. bicolor*, *L. sativum*, *C. pepo*, *S. lycopersicum*, and *L. sativa*).

Interestingly, the two species showing the most evident root hair development, *C. sativus* and *S. alba*, were also the most responsive to PFCA exposure. Despite displaying opposite response patterns, it should be noted that stimulations and inhibitions are stress responses. The mechanisms underpinning the two opposite morphological effects remain to be investigated. Disruption of hormonal processes involved in development of RSA, such as auxin-related pathways, may represent a possible explanation underlying the contrasting root responses observed in this study [15].

### 4.2. The Most Sensitive Endpoint

Overall, PFCA exposure resulted in limited effects on germination-related parameters, whereas root-related endpoints were more frequently affected, including at relatively low exposure concentrations. These results indicate that early root development represents the most sensitive indicator of PFCA-induced effects. This observation is consistent with the findings of previous studies investigating the effects of PFASs on lettuce, cucumber and other crop species. In these studies, root elongation was identified as a more responsive endpoint than germination [7,13,21,32].

The outcomes from this study and the related literature highlight that the PFAS groups are not expected to be lethal for vascular plants, while they induce stress responses even at environmentally relevant concentrations. This research suggests that such stresses may precipitate ecologically significant consequences at elevated ecological tiers. The demands of detoxification are expected to disrupt the energetic balance, thereby prompting a reallocation of resources towards maintenance and stress responses. At the population level, this shift should favour survival and growth at the expense of reproduction, ultimately reducing fitness. At broader ecological scales, such as the ecosystem level, such imbalances may have a detrimental effect on primary productivity, which can in turn lead to cascading alterations of trophic networks.

### 4.3. Carbon-Chain Length

In addition to species-specific sensitivity, plant responses to PFCA exposure varied among compounds, without following a clear linear relationship with carbon-chain length. Although carbon-chain length is known to influence PFAS uptake and translocation in plants [9,10], our results indicate that it alone does not reliably predict phytotoxicity under the tested conditions. Among the tested compounds, PFPrA, PFPeA and especially PFHpA elicited the most evident responses across species and endpoints, whereas PFBA showed the most limited response pattern, with a significant effect restricted to root stimulation in *S. alba*. PFOA also produced comparatively fewer responses, mainly affecting germination-related endpoints in selected species such as *S. bicolor* and *S. lycopersicum*. Overall, these findings suggest that compound-specific phytotoxicity cannot be explained solely by carbon-chain length.

## 5. Conclusions

This study provides a comparative assessment of PFCA phytotoxicity across multiple plant species and early developmental endpoints, showing that early plant responses are strongly species-specific and endpoint-dependent. PFCA-induced effects were mainly expressed through changes in root elongation, whereas germination showed limited sensitivity across species. The evidence obtained suggests that these PFASs are not anticipated to be lethal to vascular plants; however, they have the potential to induce stress responses even at environmentally relevant concentrations. The work needed to confront these pressures may engender a multitude of far-reaching consequences at higher levels of the ecological organization. From a pragmatic perspective, the most noteworthy finding is the considerable variation in sensitivity exhibited by the different species that were tested. In view of the data, it is proposed that cucumber (*Cucumis sativus)* and mustard *(Sinapis alba)* should be utilized as model species to evaluate the toxicity of these compounds in the soil.

## Figures and Tables

**Table 1:** Summary of significant PFCA effects on early plant development, expressed as germination inhibition (G%) and root elongation inhibition (L%), across the tested plant species. Only statistically significant responses are reported. Values indicate the PFCA concentration at which a significant effect was observed (µg kg^−1^), together with the direction of change relative to the control. For G% and L%, ↑ indicates increased inhibition, corresponding to an inhibitory effect, whereas ↓ indicates reduced inhibition, corresponding to a stimulatory effect. Asterisks indicate a significant global test applied to each PFCA within each species and endpoint: * *p* < 0.05; ** *p* < 0.01; “ns” indicates no significant effect.

Species	PFPrA	PFBA	PFPeA	PFHpA	PFOA
**G (%)**					
*Sorghum bicolor*	ns	ns	ns	ns	1 ↑*
*Cucumis sativus*	100 ↑**	ns	ns	ns	ns
*Cucurbita pepo*	ns	ns	0.01 ↑* 10 ↑*	ns	ns
*Solanum lycopersicum*	ns	ns	ns	ns	100 ↑*
**L (%)**					
*Sinapis alba*	1 ↓*	0.01 ↓*	ns	0.01 ↓** 10 ↓*	ns
*Cucumis sativus*	0.1 ↑*	ns	100 ↑*	1 ↑** 10 ↑* 100 ↑**	ns
*Lactuca sativa*	ns	ns	100 ↓*	ns	ns

**Table 2:** Summary of GAM model statistics for each PFCA in *Sinapis alba*, including Akaike’s Information Criterion (AIC), adjusted R^2^, deviance explained, estimated degrees of freedom (EDF), and *p*-value of the smooth term.

PFAS	AIC	R^2^	Deviance_Explained	EDF	*p*_Value
PFPrA	154.495	0.599	0.666	2.844	0.0017
PFBA	158.871	0.008	0.067	1	0.301
PFPeA	154.899	−0.037	0.024	1	0.538
PFHpA	156.664	−0.022	0.046	1.13	0.59
PFOA	160.153	0.31	0.398	2.173	0.0509

**Table 3:** Summary of GAM model statistics for each PFCA in *Cucumis sativus,* including Akaike’s Information Criterion (AIC), adjusted R^2^, deviance explained, estimated degrees of freedom (EDF), and *p*-values of the smooth terms.

PFCAs	AIC	R^2^	Deviance_Explained	EDF	*p*_Value
PFPrA	239.17	0.099	0.133	1	0.0567
PFBA	270.473	0.05	0.108	1.639	0.333
PFPeA	220.67	0.431	0.463	1.542	0.000578
PFHpA	162.862	0.47	0.521	1.621	0.00478
PFOA	341.355	−0.024	0.002	1	0.774

**Table 4:** The ratio between observed and predicted maximum root length (M_LLR_) across plant species. The ratio between observed and model-estimated M_LLR_ (%) is reported for each species, together with the standard deviation (SD). Predicted M_LLR_ values were derived from species-specific linear regressions fitted on control data, using mean root length (M_LR_) as the explanatory variable and M_LLR_ as the dependent variable.

Species	M_LLR_obs_/M_LLR_est_ (%)	S.D.
*Sorghum bicolor*	89	24
*Lepidium sativum*	98	11
*Cucurbita pepo*	94	25
*Lactuca sativa*	111	33
*Solanum lycopersicum*	90	23
*Sinapis alba*	104	11
*Cucumis sativus*	101	14

## Data Availability

The original contributions presented in this study are included in the article/Supplementary Materials. Further inquiries can be directed to the corresponding author.

## Supplementary Materials

The following supporting information can be downloaded at https://www.mdpi.com/article/10.3390/toxics14060455/s1. Table S1: Germination (G). mean root length (M_LR_), Table S2: Summary of PFCAs effects on early plant development expressed as Germination Index, Figure S1: L% of *S. alba*, Figure S2: L% of *C. sativus*, Figure S3: Seedling morphology.
