# Supplementary material for: Root Growth as an Early Indicator of PFAS Phytotoxicity in Plants
Source: Toxics. 2026 May 22;14(6):455. doi: 10.3390/toxics14060455 (PMC13306679; doi:10.3390/toxics14060455)
Supplement: Supplementary file 1 [file toxics-14-00455-s001.zip › toxics-4319589-supplementary.pdf]

**SUPPLEMENTARY MATERIALS**

# Root growth as an early indicator of PFAS phytotoxicity in plants

Lara Nigro[1]. Lorenzo Federico[1*]. Valeria Tatangelo[1]. Sara Villa[1]

[1]DISAT. Department of Earth and Environmental Sciences. University of Milano-Bicocca. Piazza della Scienza 1. Milan 20126. Italy.
*corresponding author: Lorenzo.federico@unimib.it

## 3 RESULTS

### *3.1 Overall results*

| *Species* | ***S. bicolor*** | | | | | | | | | | | | | | | | | | | | | | | | | |
|---|---|---|---|---|---|---|---|---|---|---|---|---|---|---|---|---|---|---|---|---|---|---|---|---|---|---|
| *PFCA* | *Ctrl* | *PFPrA* | | | | | *PFBA* | | | | | *PFPeA* | | | | | *PFHpA* | | | | | *PFOA* | | | | |
| *Conc. (µg/kg)* | *0* | *0.01* | *0.1* | *1* | *10* | *100* | *0.01* | *0.1* | *1* | *10* | *100* | *0.01* | *0.1* | *1* | *10* | *100* | *0.01* | *0.1* | *1* | *10* | *100* | *0.01* | *0.1* | *1* | *10* | *100* |
| *G (%)* | *93* | *77* | *68* | *77* | *70* | *57* | *77* | *77* | *87* | *73* | *70* | *67* | *73* | *87* | *77* | *83* | *80* | *63* | *80* | *63* | *97* | *77* | *83* | ***57**** | *70* | *67* |
| *MLR (mm)* | *11.9* | *13.9* | *13.1* | *15.2* | *11.5* | *17.1* | *16* | *15.9* | *14.9* | *15.6* | *10.9* | *16.8* | *14.2* | *13* | *13.2* | *12.4* | *17* | *14.7* | *12.5* | *16.2* | *14.1* | *12.6* | *13* | *10.9* | *13.6* | *14.1* |
| *MLLR (mm)* | *23.2* | *23.4* | *21.9* | *25.9* | *20.1* | *28.7* | *27.8* | *24.2* | *25.6* | *27.1* | *18.7* | *33.4* | *25.7* | *21.8* | *26.4* | *22.1* | *29.1* | *22.3* | *22.8* | *24.3* | *24.2* | *25.3* | *20.5* | *20.4* | *20.1* | *22.5* |

| *Species* | ***L. sativum*** | | | | | | | | | | | | | | | | | | | | | | | | | |
|---|---|---|---|---|---|---|---|---|---|---|---|---|---|---|---|---|---|---|---|---|---|---|---|---|---|---|
| *PFCA* | *Ctrl* | *PFPrA* | | | | | *PFBA* | | | | | *PFPeA* | | | | | *PFHpA* | | | | | *PFOA* | | | | |
| *Conc. (µg/kg)* | *0* | *0.01* | *0.1* | *1* | *10* | *100* | *0.01* | *0.1* | *1* | *10* | *100* | *0.01* | *0.1* | *1* | *10* | *100* | *0.01* | *0.1* | *1* | *10* | *100* | *0.01* | *0.1* | *1* | *10* | *100* |
| *G (%)* | *100* | *90* | *90* | *73* | *90* | *83* | *93* | *93* | *80* | *97* | *87* | *93* | *100* | *77* | *87* | *97* | *93* | *97* | *80* | *97* | *97* | *100* | *90* | ***73**** | *87* | *100* |
| *MLR (mm)* | *60.5* | *56.1* | *62.3* | *59.4* | *62.2* | *59.2* | *58.6* | *61.7* | *62.4* | *61* | *64.2* | *59.2* | *65.8* | *59.5* | *60* | *63.5* | *54.3* | *59.2* | *58.4* | *65.2* | *59.4* | *65.8* | *66.5* | *61.4* | *66.8* | *60.7* |
| *MLLR (mm)* | *84.6* | *75.5* | *84.7* | *84.6* | *82.2* | *75.2* | *89* | *83.9* | *83.1* | *83* | *85.2* | *82* | *87.3* | *81.5* | *80.9* | *84.2* | *82.7* | *80.6* | *86.8* | *81.8* | *78.7* | *93.1* | *82.6* | *80.9* | *85.4* | *92.2* |
| *Species* | ***S. alba*** | | | | | | | | | | | | | | | | | | | | | | | | | |
| *PFCA* | *Ctrl* | *PFPrA* | | | | | *PFBA* | | | | | *PFPeA* | | | | | *PFHpA* | | | | | *PFOA* | | | | |
| *Conc. (µg/kg)* | *0* | *0.01* | *0.1* | *1* | *10* | *100* | *0.01* | *0.1* | *1* | *10* | *100* | *0.01* | *0.1* | *1* | *10* | *100* | *0.01* | *0.1* | *1* | *10* | *100* | *0.01* | *0.1* | *1* | *10* | *100* |
| *G (%)* | *100* | *100* | *100* | *100* | *97* | *93* | *97* | *100* | *100* | *97* | *97* | *100* | *97* | *100* | *97* | *100* | *100* | *97* | *97* | *100* | *100* | *93* | *100* | *100* | *90* | *100* |
| *MLR (mm)* | *52.9* | *57.8* | *61.1* | ***82.2 ***** | *72.2* | *73.6* | ***74.3 **** | *67.3* | *71.4* | *61.5* | *59.7* | ***62.4 **** | *66* | *67.8* | *61.2* | *65.2* | ***74.4**** | *64.7* | *63.5* | ***72.1 **** | *65.7* | *66.8* | *64.9* | *70.9* | *77.7* | *57.7* |

| | | | | | | | | | | | | | | | | | | | | | | | | | | |
|---|---|---|---|---|---|---|---|---|---|---|---|---|---|---|---|---|---|---|---|---|---|---|---|---|---|---|
| *MLLR (mm)* | *78.8* | *78.9* | *85.4* | ***119.2**** | *105.1* | *98.7* | *95.5* | *104* | *105.1* | *97* | *88.8* | *94.1* | *92.4* | *91.6* | *90.9* | *94.6* | ***104.9**** | *93* | *87.2* | *94.1* | *98.8* | *101.7* | ***104.6**** | *102* | *99.7* | *83.1* |
| *Species* | ***C. sativus*** | | | | | | | | | | | | | | | | | | | | | | | | | |
| *PFCA* | *Ctrl* | *PFPrA* | | | | | *PFBA* | | | | | *PFPeA* | | | | | *PFHpA* | | | | | *PFOA* | | | | |
| *Conc. (µg/kg)* | *0* | *0.01* | *0.1* | *1* | *10* | *100* | *0.01* | *0.1* | *1* | *10* | *100* | *0.01* | *0.1* | *1* | *10* | *100* | *0.01* | *0.1* | *1* | *10* | *100* | *0.01* | *0.1* | *1* | *10* | *100* |
| *G (%)* | *100* | *96* | *100* | *100* | *83* | *88* | *96* | *94* | *100* | *96* | *96* | *96* | *96* | *100* | *100* | *96* | *100* | *100* | *91* | *88* | *96* | *100* | *100* | *92* | *100* | *100* |
| *MLR (mm)* | *41.8* | *52.3* | *51.3* | *49.3* | *39.3* | *39.3* | *52.8* | *53.8* | *44.3* | *31* | *42.1* | *46.3* | *43.1* | *36.2* | *30.2* | ***27.4**** | *57.9* | *41.1* | ***27**** | ***32.1**** | ***24.6***** | *61.5* | *55* | *49.5* | *33.5* | *32.5* |
| *MLLR (mm)* | *53.2* | *68.5* | *63.3* | *62.8* | *49.7* | *53.3* | *67.3* | *73.4* | *63.3* | *57.3* | *55.3* | *58.7* | *56.1* | *46.4* | *45.6* | ***37.2**** | *71.3* | *56.4* | *41.6* | *49.2* | ***32.3**** | *85.9* | *72.5* | *65.7* | *43.9* | *45* |
| *Species* | ***C. pepo*** | | | | | | | | | | | | | | | | | | | | | | | | | |
| *PFCA* | *Ctrl* | *PFPrA* | | | | | *PFBA* | | | | | *PFPeA* | | | | | *PFHpA* | | | | | *PFOA* | | | | |
| *Conc. (µg/kg)* | *0* | *0.01* | *0.1* | *1* | *10* | *100* | *0.01* | *0.1* | *1* | *10* | *100* | *0.01* | *0.1* | *1* | *10* | *100* | *0.01* | *0.1* | *1* | *10* | *100* | *0.01* | *0.1* | *1* | *10* | *100* |
| *G (%)* | *70* | *50* | *50* | *53* | *43* | *33* | *57* | *63* | *67* | *50* | *53* | ***40**** | *53* | *53* | *43** | *60* | *47* | *40* | *60* | *47* | *53* | *47* | *47* | *47* | *47* | *47* |

| | | | | | | | | | | | | | | | | | | | | | | | | | | |
|---|---|---|---|---|---|---|---|---|---|---|---|---|---|---|---|---|---|---|---|---|---|---|---|---|---|---|
| *MLR (mm)* | *10.9* | *13.2* | *13.1* | *13.4* | *12* | *10.5* | *6.3* | *16.8* | *11.9* | *9.3* | *10.1* | *13.5* | *11.6* | *8.4* | *10.6* | *12* | *9.2* | *13.4* | *11.2* | *14.4* | *13.8* | *8.1* | *9.8* | *12.3* | *16* | *12.6* |
| *MLLR (mm)* | *21.1* | *21.6* | *23.9* | *21.5* | *22.7* | *14.5* | *14.6* | *23.2* | *22.9* | *17* | *18.1* | *17.4* | *20.2* | *15.5* | *16.7* | *22.8* | *15.5* | *24* | *20.4* | *22.4* | *27.6* | *15.3* | *14.9* | *25.3* | *24.7* | *22.9* |
| *Species* | ***L. sativa*** | | | | | | | | | | | | | | | | | | | | | | | | | |
| *PFCA* | *Ctrl* | *PFPrA* | | | | | *PFBA* | | | | | *PFPeA* | | | | | *PFHpA* | | | | | *PFOA* | | | | |
| *Conc. (µg/kg)* | *0* | *0.01* | *0.1* | *1* | *10* | *100* | *0.01* | *0.1* | *1* | *10* | *100* | *0.01* | *0.1* | *1* | *10* | *100* | *0.01* | *0.1* | *1* | *10* | *100* | *0.01* | *0.1* | *1* | *10* | *100* |
| *G (%)* | *93* | *100* | *93* | *97* | *93* | *93* | *97* | *97* | *100* | *93* | *100* | *93* | *90* | *83* | *97* | *90* | *93* | *87* | *97* | *97* | *93* | *100* | *93* | *87* | *97* | *93* |
| *MLR (mm)* | *22.2* | *22.8* | *21.7* | *22.4* | *22.1* | *24* | *21.2* | *36.6* | *21.2* | *22.7* | *23.1* | *20.7* | *22.8* | *23.4* | *21* | ***26.1**** | *23.7* | *21.6* | *27.1* | *21.9* | *21.7* | *24.2* | *25.6* | *21.3* | *20.6* | *23.6* |
| *MLLR (mm)* | *28.9* | *30.7* | *27.9* | *28.5* | *29.2* | *31.6* | *27.2* | *45* | *30.5* | *29.1* | *27.9* | *31.3* | *28.5* | *31.1* | *28.9* | *34* | *30.2* | *28.7* | *34.3* | *33.1* | *31.1* | *31* | *30.6* | *30.3* | *34.6* | *29.5* |
| *Species* | ***S. lycopersicum*** | | | | | | | | | | | | | | | | | | | | | | | | | |
| *PFCA* | *Ctrl* | *PFPrA* | | | | | *PFBA* | | | | | *PFPeA* | | | | | *PFHpA* | | | | | *PFOA* | | | | |
| *Conc. (µg/kg)* | *0* | *0.01* | *0.1* | *1* | *10* | *100* | *0.01* | *0.1* | *1* | *10* | *100* | *0.01* | *0.1* | *1* | *10* | *100* | *0.01* | *0.1* | *1* | *10* | *100* | *0.01* | *0.1* | *1* | *10* | *100* |

| | | | | | | | | | | | | | | | | | | | | | | | | | |
|---|---|---|---|---|---|---|---|---|---|---|---|---|---|---|---|---|---|---|---|---|---|---|---|---|---|
| *G (%)* | 80 | 57 | 80 | 90 | 83 | 87 | 70 | 80 | 73 | 83 | 80 | 73 | 53 | 73 | 77 | 80 | 83 | 67 | 83 | 93 | 57 | 60 | 67 | 70 | 60 | **47*** |
| *MLR (mm)* | 71.7 | 63.8 | 84.5 | 72 | 69.7 | 79.9 | 87.3 | 64.6 | 73 | 76.1 | 96.4 | 88 | 70.1 | 78.1 | 91.5 | 85.1 | 84.5 | 84.9 | 78.6 | 85.6 | 91.1 | 74.3 | 78.6 | 78.9 | 73.9 | 84.2 |
| *MLLR (mm)* | 119.3 | 99.2 | 117 | 122.7 | 104.5 | 121.7 | 140.1 | 104 | 126.7 | 112 | 127.3 | 135.4 | 109 | 110.2 | 126 | 153.4 | 120.9 | 129 | 107 | 128.7 | 116.4 | 98.5 | 108.2 | 128 | 101.3 | 120 |

Table S1 Germination (G). mean root length ($M_{LR}$). maximum root length ($M_{LLR}$) of different plant species exposed to increasing concentrations (0.01–100 $\mu g\ kg^{-1}$ d.w.) of selected PFCAs (PFPrA. PFBA. PFPeA. PFHpA. PFOA). * indicate statistically significant differences compared with the control. based on ANOVA followed by pairwise post-hoc comparisons. or Kruskal–Wallis followed by pairwise comparisons when parametric assumptions were not met ($p < 0.05$; $p < 0.01$; $p < 0.001$).

| Species | PFPrA | PFBA | PFPeA | PFHpA | PFOA |
|---|---|---|---|---|---|
| | | | GI (%) | | |
| *Sorghum bicolor* | ns | ns | ns | ns | ns |
| *Lepidium sativum* | ns | ns | ns | ns | ns |
| *Sinapis alba* | 1 ↑* | ns | ns | 0.01 ↑* | ns |
| *Cucumis sativus* | 0.1 ↑** | ns | ns | 1 ↓** 10 ↓* 100 ↓** | ns |
| *Cucurbita pepo* | ns | ns | 1 ↓*10↓* | ns | ns |
| *Lactuca sativa* | ns | ns | ns | ns | ns |
| *Solanum lycopersicum* | ns | ns | ns | ns | ns |

Table S2. Summary of PFCAs effects on early plant development expressed as Germination Index (GI%, APAT/IRSA-UNICHIM) across the tested plant species. Values indicate the PFCA concentration at which a significant effect was observed (μg $kg^{-1}$), together with the direction of change relative to the control. ↑ indicates increased GI%, whereas ↓ indicates reduced GI%. Asterisks indicate significant post-hoc differences versus the control (* $p < 0.05$; ** $p < 0.01$). “ns” indicates no significant effect.

*3.3 Species with Highest Sensitivity to PFCAs*

*3.3.1 S. alba*

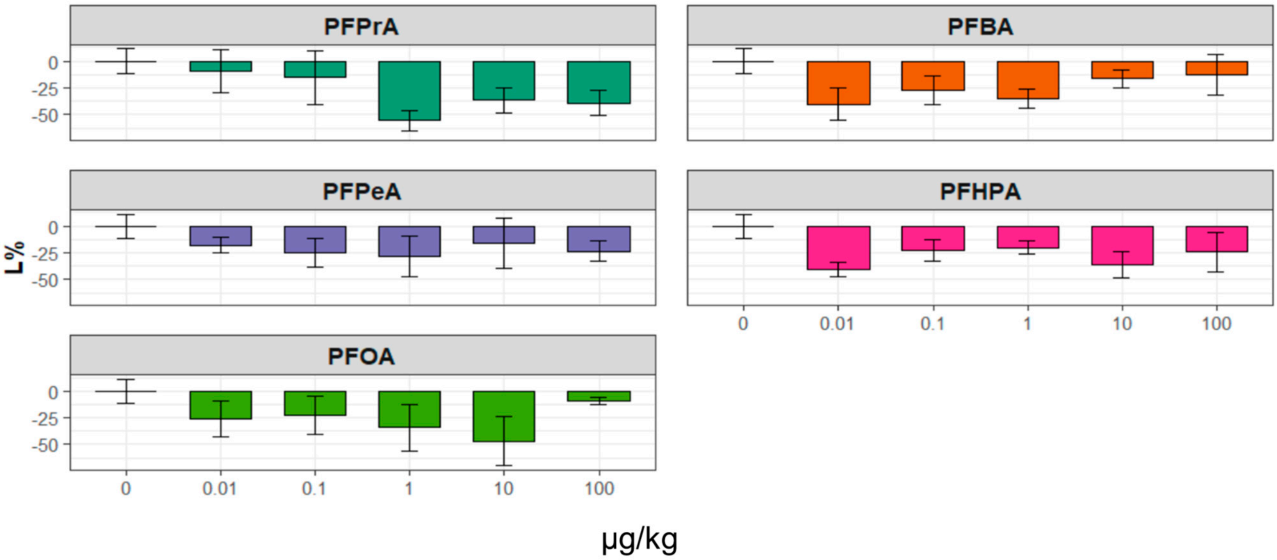


Figure S1 L% of *S. alba* seedlings exposed to increasing concentrations (0.01–100 µg $kg^{-1}$ d.w.) of perfluoropropionic acid (PFPrA) . perfluorobutanoic acid (PFBA). perfluoropentanoic acid (PFPeA). perfluoroheptanoic acid (PFHpA). perfluorooctanoic acid (PFOA). Different letters indicate statistically significant differences among treatments according to Tukey's HSD test ($p < 0.05$) exposure in triplicate (n=3 per treatments).

*3.3.2 C. sativus*

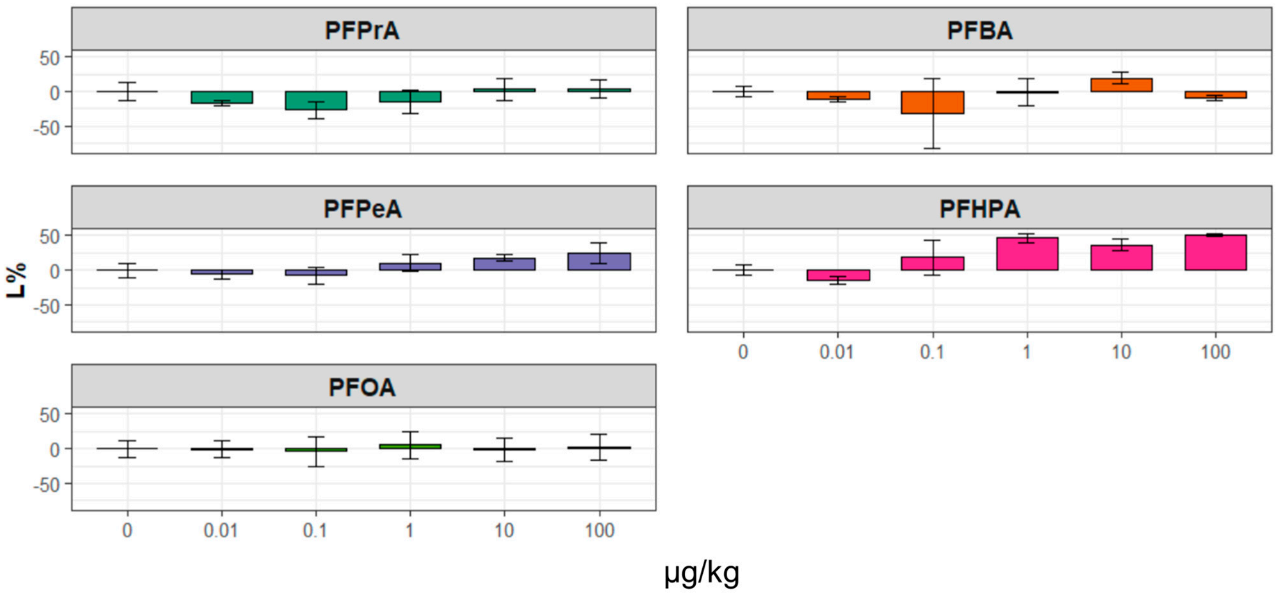


Figure S2 L% of *C. sativus* seedlings exposed to increasing concentrations (0.01–100 µg $kg^{-1}$ d.w. of perfluoropropionic acid (PFPrA) . perfluorobutanoic acid (PFBA). perfluoropentanoic acid (PFPeA). perfluoroheptanoic acid (PFHpA). perfluorooctanoic acid (PFOA). Different letters indicate statistically significant differences among treatments according to Tukey's HSD test ($p < 0.05$) exposure in triplicate (n=3 per treatments).

## *4 DISCUSSION*

### *4.1 Species-specific sensitivity to PFCAs*

| Species | Picture at the end of the experiment | Description of root system architecture | Bibliography |
|---|---|---|---|
| ***S. bicolor*** | 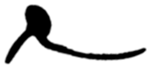 | At 72 h post-germination, the caryopsis remains attached and partially intact. The seedling exhibits a single seminal root emerging from the basal side of the seed and an emerging shoot enclosed within the coleoptile. Root hairs may already be present along the differentiation zone of the seminal root, as reported in sorghum seedlings as early as 60 h after germination Root hairs were not clearly observable under our experimental conditions | Singh et al. 2010<br>Yang et al., 2004 |
| ***L. sativum*** | 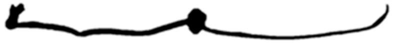 | At 72 h post-germination, seedlings exhibited a long primary root, an elongated hypocotyl and emerging cotyledons, consistent with the early seedling morphology described for this species. Root hairs were not clearly observable under our experimental conditions. | Bosker et al., 2019 |
| ***S. alba*** | 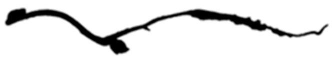 | Germination in *Sinapis alba* is epigeal. During the first 72 h post-germination, the radicle rapidly emerges from the seed and develops into the primary root, which undergoes vigorous elongation. Concurrently, the hypocotyl elongates and elevates the cotyledons above the substrate. Root hairs become visible along the elongating primary root during this early stage | Jan et al., 2024 |
| ***C. sativus*** | 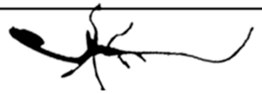 | *Cucumis sativus* seedlings develop a dominant elongating primary root bearing root hairs, together with an elongated hypocotyl and emerging cotyledons. Early lateral roots are also visible near the basal region of the primary root. | Liu et al., 2021 |
| ***C. pepo*** | 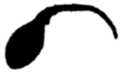 | During early seedling development, primary root elongation predominates in *Cucurbita pepo*. At approximately 72 h post-germination, seedlings exhibit an elongating primary root and an emerging hypocotyl, while root hairs are not yet clearly visible along the root surface, as these typically develop later in the root differentiation zone. | Bewley et al., 2012 |
| ***L. sativa*** | 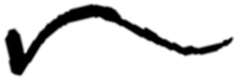 | Germination in *Lactuca sativa* is epigeal. The radicle emerges first from the seed and develops into the primary root, while hypocotyl elongation elevates the cotyledons above the substrate. At approximately 72 h post-germination, seedlings show an elongating primary root and a developing hypocotyl, while lateral roots are typically absent or only beginning to initiate. | Bewley et al., 2012;<br>Taiz et al., 2015 |
| ***S. lycopersicum*** | 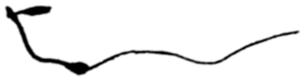 | During early seedling development, *Solanum lycopersicum* forms an elongating primary root that becomes organized into distinct zones, including a differentiation zone where root hairs emerge. At approximately 1 week, seedlings display an elongating primary root and a curved hypocotyl, while lateral roots are not yet developed. | Grierson & Schiefelbein 2002 |

Figure S3 Representative images of early seedling morphology in the tested plant species under control conditions. Images highlight qualitative differences in early root system architecture, including primary root development and root hair visibility among species. These observations were used as descriptive support for the interpretation of species-specific responses to PFCA exposure.

Singh, V., van Oosterom, E. J., Jordan, D. R., Messina, C. D., Cooper, M., & Hammer, G. L. (2010). Morphological and architectural development of root systems in sorghum and maize. *Plant and Soil*, *333*(1), 287-299.

Yang, X., Scheffler, B. E., & Weston, L. A. (2004). SOR1, a gene associated with bioherbicide production in sorghum root hairs. *Journal of Experimental Botany*, *55*(406), 2251-2259.

Bosker, T., Bouwman, L. J., Brun, N. R., Behrens, P., & Vijver, M. G. (2019). Microplastics accumulate on pores in seed capsule and delay germination and root growth of the terrestrial vascular plant Lepidium sativum. *Chemosphere*, *226*, 774-781.

Jan, M., Muhammad, S., Jin, W., Zhong, W., Zhang, S., Lin, Y., ... & Wang, G. (2024). Modulating root system architecture: cross-talk between auxin and phytohormones. *Frontiers in Plant Science*, *15*, 1343928.

Liu, X., Chen, J., & Zhang, X. (2021). Genetic regulation of shoot architecture in cucumber. *Horticulture Research*, *8*.

Bewley, J. D., Bradford, K., & Hilhorst, H. (2012). *Seeds: physiology of development, germination and dormancy*. Springer Science & Business Media.

Taiz, L., Zeiger, E., Møller, I. M., & Murphy, A. (2015). Plant physiology and development.

Grierson, C., & Schiefelbein, J. (2002). Root hairs. *The Arabidopsis book/American Society of Plant Biologists*, *1*, e0060.
